# Cytokine changes associated with the maternal immune activation (MIA) model of autism: A penalized regression approach

**DOI:** 10.1371/journal.pone.0231609

**Published:** 2020-08-06

**Authors:** Cristina Paraschivescu, Susana Barbosa, Thomas Lorivel, Nicolas Glaichenhaus, Laetitia Davidovic

**Affiliations:** 1 Centre National de la Recherche Scientifique, Institut de Pharmacologie Moléculaire et Cellulaire, Valbonne, France; 2 Université Côte d’Azur, Nice, France; Universidade do Estado do Rio de Janeiro, BRAZIL

## Abstract

Maternal immune activation (MIA) during pregnancy induces a cytokine storm that alters neurodevelopment and behavior in the progeny. In humans, MIA increases the odds of developing neuropsychiatric disorders such as autism spectrum disorder (ASD). In mice, MIA can be induced by injecting the viral mimic polyinosinic:polycytidylic acid (poly(I:C)) to pregnant dams. Although the murine model of MIA has been extensively studied, it is not clear whether MIA results in cytokine changes in the progeny at early postnatal stages. Further, the murine model of MIA suffers from a lack of reproducibility and high inter-individual variability. Multivariable (MV) statistical analysis is widely used in human studies to control for confounders and covariates such as sex, age and exposure to environmental factors. We therefore reasoned that animal studies in general and studies on the MIA model in particular could benefit from MV analyses to account for complex phenotype interactions and high inter-individual variability. Here, we used MV statistical analysis to identify cytokines associated with MIA after adjustment for covariates. Besides confirming the association between previously described variables and MIA, we identified new cytokines that could play a role in behavioural alterations in the progeny during the early postnatal period.

## 1. Introduction

A large body of evidence suggests that neurodevelopment before and after birth, as well as behaviour during the postnatal period are regulated by cytokines. First, cytokine levels in both blood and brain are tightly regulated during the prenatal and postnatal periods [1, 2]. Second, cell culture experiments using both human and rodent cells have shown that cytokines regulate neurogenesis, neuronal migration, synaptogenesis, synaptic pruning, angiogenesis, myelination and apoptosis *in vitro* [3, 4]. Third, mice deficient in cytokine or cytokine receptor genes often exhibit behavioural alterations [4]. Fourth, maternal immune activation (MIA) during pregnancy increases the odds of neuropsychiatric disorders such as autism spectrum disorders (ASD) [5, 6]. Further, elevated levels of several cytokines including interferon (IFN)-γ, interleukin (IL)-1α, IL-4, IL-5 or IL-6 in maternal blood during pregnancy are associated with increased ASD risk [7, 8]. Also, elevated levels of IL-1β, IL-4, IL-6 or IL-8 in dried blood spots collected after birth increase the risk of later ASD diagnosis [9, 10]. In rodents, MIA can be modelled by injecting poly(I:C) to pregnant dams [11–13]. Poly(I:C) induces a systemic inflammatory response characterized by increased serum levels of maternal proinflammatory cytokines, which provokes irreversible neurodevelopmental defects in the foetus [14], resulting in an altered behaviour in the progeny, and notably social behaviour and communication deficits reminiscent of ASD symptoms [11–13]. Further studies have demonstrated that these behavioural alterations are notably dependent on IL-6 and IL-17A induced by MIA during gestation [15–17]. While MIA-induced changes in cytokine levels during the postnatal period may also impact neurodevelopment, this issue has not been extensively investigated. The rodent models of MIA have allowed for a better understanding of the role of maternal and foetal cytokines in neurodevelopment. However, recent studies have pointed that the MIA model presents reproducibility issues across different laboratories as well as within each laboratory [18, 19]. This lack of consistency could be accounted for by non-documented differences in experimental conditions such as housing parameters, timing, intensity and duration of MIA, which appear critical to determine the nature of brain and behavioural alterations observed in progeny [18, 19]. However, inconsistency may also be explained by complex interactions between biological and environmental factors yielding high inter-individual variability in the neurodevelopmental response to MIA.

In contrast to human studies in which individuals are both genetically different and exposed to different environmental conditions during both the prenatal and postnatal periods, most animal studies involve groups of genetically homogeneous individuals that have been bred and housed in the same environment. As a result, inter-individual differences in animal studies are often relatively low and univariate statistical analysis such as parametric (Student’s t-test, ANOVA) and non-parametric (Mann-Whitney test, Kruskal-Wallis) tests is usually the gold standard to compare different experimental groups. In contrast, multivariable (MV) statistical analysis is routinely used in human studies to control for covariates and confounders including sex, age and exposure to environmental factors. Following this line of thoughts, we reasoned that MV statistical analysis might prove helpful to identify cytokines associated with MIA in the postnatal period.

## 2. Materials and methods

### 2.1. Animals

Animal housing and experimentation were conducted in facilities certified by regional authorities (Direction Départementale de Protection des Populations des Alpes-Maritimes, accreditation #C-06-152-5). The procedures involved in this study were approved by the local ethics committee for animal experimentation (Ciepal Azur) and the Ministère de l’Enseignement Supérieur et de la Recherche, in agreement with the EU Directive 2010/63/EU for animal experiments. Given the recent demonstration that C57Bl/6N Taconic mice were more susceptible to MIA-induced behavioural defects [16], twelve female and four male C57Bl/6N founder mice were purchased from Taconic Biosciences (Lille Skensved, Denmark). Mice were housed in open medium cages equipped with wooden litter, cotton to nidify as well as a one plastic house, in a temperature (22–24°C) and hygrometry (70–80%)-controlled room, with a 12-h light/dark cycle (lights on from 8:00 a.m. to 8:00 p.m.) with food (standard chow, reference 4RF25, Mucedola) and water *ad libitum*. Mice were housed by 3–5 animals per cage.

### 2.2. Experimental procedures

The MIA model was generated according to recommended guidelines [18, 19]. Females were mated with males for 16h (6 p.m.-10 a.m. next day, considered embryonic day (E) 0.5), by groups of 3 females for 1 male. After mating, females were left undisturbed, with the exception of weekly cage cleaning. Pregnant dams (identified based on minimal body mass gain of 3 g between E0.5-E11.5) were randomly assigned to the experimental groups and injected with poly(I:C) (n = 13) or vehicle (saline, n = 8) at E12.5 between 9–10 p.m. Due to previously reported intra-lot variability of poly(I:C) [13, 18], a single lot (reference P9582, Sigma-Aldrich) was dissolved in sterile double-distilled water at room temperature (RT) to generate a stock solution based on pure poly(I:C) mass (20μg/μL). Stock solution was aliquoted and stored at -20°C until use. For each cohort, a new aliquot was diluted in 0.9% NaCl to the working concentration of 1μg/μL. Pregnant dams received a single intraperitoneal injection of either poly(I:C) at a dose of 5 mg/kg (5μL/g body mass of the 1μg/μL solution) or 5μL/g body mass of saline.

### 2.3. Collected variables

#### 2.3.1. Biological variables

Maternal and paternal ages were recorded. Maternal rectal temperature was recorded before, as well as 3 and 6 h post injection of Saline or poly(I:C). The maternal Δtemperature was calculated by subtracting the temperature 3 h post injection to the temperature before injection. Body mass was recorded before and 24 h post injection. The maternal Δmass was calculated by subtracting the body mass 24 h post injection to the body mass before injection. At E16.5, the pregnant dams were isolated into individual cages until birth, considered as postnatal day 0 (P0) and left undisturbed until P4. Litter size was recorded at P4. Only males were included in the study, but females remained in the litter to preserve numbers and sex balance. Male pups were individually marked at P4. We followed a total of n = 27 male pups born from mothers injected with saline (from 8 litters spread across 3 independent cohorts) and n = 40 male pups born from mothers injected with poly(I:C) (from 12 litters spread across 4 independent cohorts).

Pups were weighted at P15 and euthanized using a lethal dose of anaesthetic. Blood was collected by cardiac puncture into 1.5 ml Eppendorf tubes, allowed to clot for 30 min RT and centrifuged (10,000g, 4°C, 20 min) to recover serum stored at -80°C. The V-PLEX® Mouse Cytokine 29-Plex Kit electrochemiluminescence-based assays (Meso Scale Diagnostics, Rockville, USA) was used to measure IFN-γ, IL-1β, IL-2, IL-4, IL-5, IL-6, IL-12p70, CXCL1, TNF-α, IL-9, CCL2, IL-33, IL-27p28/IL-30, IL-15, IL-17A/F, CCL3, CXCL10, CXCL2, CCL20, IL-22, IL-23, IL-17C; IL-31, IL-21, IL-16, IL-17A, IL-17E/IL-25. Assays were performed according to the manufacturer’s instructions. Cytokines (IL-2, IL-4, IL-12p70, IL-9, IL-17A/F, IL-22, IL-23, IL-17C, IL-31, IL-21, IL-17E and IL-25) below the Lower Limit of Detection (LLOD) in more than 20% of the samples were excluded. For the other cytokines, cytokine levels below LLOD were imputed a value equal to half the LLOD value indicated by the manufacturer.

#### 2.3.2. Behavioural variables

Pups were isolated from their mother and from each other at P6, and placed on a cotton-padded dish into a thermo-controlled (26°C) soundproof chamber. Ultrasonic vocalizations (USV) were recorded for 5 min using the UltraSoundGate Condenser Microphone and 116 USB Audio device (Avisoft Bioacoustics), as described [20, 21]. Four pups were recorded simultaneously in parallel chambers. Sonograms were analysed with the AvisoftSASLab Pro software (version 5.2.12, Avisoft Bioacoustics) based on automated recognition of USV using a 25 kHz cut-off frequency and a 2–7 ms element separation, as described in [20]. Misidentified USV were manually curated and number and duration of USV were automatically extracted. At P13, pup was placed in a clean individual cage and its movements recorded during 10 min with a digital camera. Videos were analysed using the ANY-maze video-tracking software (Dublin, Ireland), which extracted the total distance travelled and the time spent mobile for each individual.

### 2.4. Statistics

#### 2.4.1. Correlation studies

Pairwise correlations between numerical variables and associated p-values were assessed using the Spearman's r correlation coefficient rank test. We did not adjust the p-values for multiple testing as this correlation analysis was mainly descriptive to highlight possible relationship between variables (multi-collinearity) and not meant for biological interpretation.

#### 2.4.2. Penalized regression

*Rationale for the choice of penalized regression*. Regression problems with many potential candidate and adjustment variables usually require model selection to find a simpler model, while keeping a good performance. While penalized regression methods are mostly used in high-dimensional settings, their usefulness in low-dimensional settings has been demonstrated [22]. Indeed, traditional stepwise selection methods, such as forward and backward selection, suffer from high variability and low accuracy, in particular in settings with large number of covariables or when there is correlation between covariables [23]. Penalized methods, such as LASSO, Elastic Net and Ridge regressions are an alternative to such traditional methods. Unlike ordinary least squares (OLS) estimation, these methods estimate the regression coefficients by minimizing the residual sum of squares (RSS), while placing a constraint on the size of the regression coefficients. This constraint or penalty has the disadvantage of biasing the coefficient estimates, however, it improves the overall prediction error of the model by decreasing the variance of the coefficient estimates or odd-ratios (OR).

A penalized regression method yields a sequence of models, each associated with specific values of the α and λ hyperparameters. α accounts for the relative importance of the L1 (LASSO) and L2 (ridge) regularizations and λ controls the magnitude of regularization [24]. Thus, a tuning method for one or both hyperparameters needs to be specified to achieve an optimal model. There are several tuning methods such as AIC, Cp statistic, average square error on the validation data and cross-validation. By minimizing the RSS using a penalty on the size of the regression coefficients, some regression coefficients will shrink towards zero. If the penalty is extreme, regression coefficients are set to zero exactly. Thus, penalized regression performs both variable selection and coefficients regularization, enhancing the prediction accuracy and interpretability of the resulting statistical models [25].

*Implementing penalized regression*. *Dataset*: All variables collected for each individual pup involved in the study were gathered in a matrix of n = 67 rows corresponding to the 67 pups and p = 26 column corresponding to the 26 variables collected (S1 Table). Penalized regression does not allow for missing values. In this study, we only considered individuals for which all variables were available. In some cases, to avoid discarding individual samples presenting missing values, imputation of missing values can be achieved using for example the Multiple Imputation by Chained Equations (MICE) package in R [26]. As recommended, for a fair comparison of the relative predictor importance across all explanatory variables, all numerical variables were mean-centered and then scaled such that the input matrix has all column-means equal to 0 and column-variances equal to 1 [27]. Categorical variables were encoded as numbers, with each category represented with a binary vector that contains 1 and 0, denoting the inclusion in one category or the other. The number of vectors depends on the number of levels for the categorical variable and full encoding means all levels are included in the analysis. In our case, the categorical variables considered were binary: multiparity (No/Yes) and the outcome (Control/MIA).

*Resampling and penalized regression*: To model the association between parental and pups variables and the outcome, i.e. being born from a poly(I:C)-injected mother”, we implemented a penalized regression (Lasso model) using the glmnet and caret R packages [24, 28]. Of note, other software is available (e.g. PROC GLMSELECT in SAS). Considering that current penalized regression methods do not provide valid confidence intervals, or p-values, for testing the significance of coefficients, we used the non-parametric bootstrap for inference [27]. The bootstrap step involved 500 resamplings of the dataset to create 500 different samples of the same size. For each of the resampling runs, we obtained the list of the variables selected by Lasso logistic regression model and the corresponding OR. Based on these, we computed for each variable: 1) the variable inclusion probability (VIP), as the percentage of the 500 bootstrap resamples in which each variable was selected by the penalized regression; 2) the mean OR, computed across the 500 bootstrap resamples, and 3) the non-parametric confidence intervals (CI). The CI was determined by first ordering the bootstrap penalized coefficient estimates from the lowest to highest, then selecting values at the chosen percentile for the confidence interval. For a confidence interval of 95%, as the lower bound we selected the OR value at the 2.5% percentile and as the upper bound of the CI the 97.5% percentile [29].

*Interpretation of VIP and coefficients*: We used the VIP as a measure of the stability of the association between the variable of interest and the outcome. Depending on the study, determining an appropriate threshold for the VIP can be challenging. In their seminal paper, Bunea et al. 2011 recommended to use a “conservative threshold of 50%” because their goal was “not to miss any possibly relevant predictors” [27]. However, this 50% threshold increases the risk of false positives. In this study, we chose to use a stringent VIP threshold of 80% to decide whether the variable under scrutiny was associated with the outcome. Then, we used the mean OR for the selected variables as a measure of the strength of the association between the variable and the outcome. The OR is calculated as the exponential function of each logistic regression coefficient. The OR represents the odds that an outcome will occur given a particular variable, compared to the odds of the outcome occurring in the absence of that variable [30]. In this study, we used the mean OR to determine whether a particular variable is associated with MIA, and to compare the magnitude of various variables for that outcome. An OR > 1 is indicative of a variable positively associated with MIA while an OR < 1 can be interpreted as a negative association between the variable an MIA. Finally, the smaller the OR 95% CI is, the better the estimation of the effect of the variable on the outcome is.

**Fig 1:**
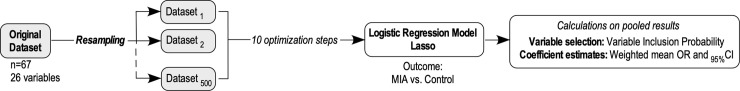
Methodological workflow for the multivariable analysis.

Fig 1 depicts the workflow of the analysis.

## 3. Results

**Fig 2:**
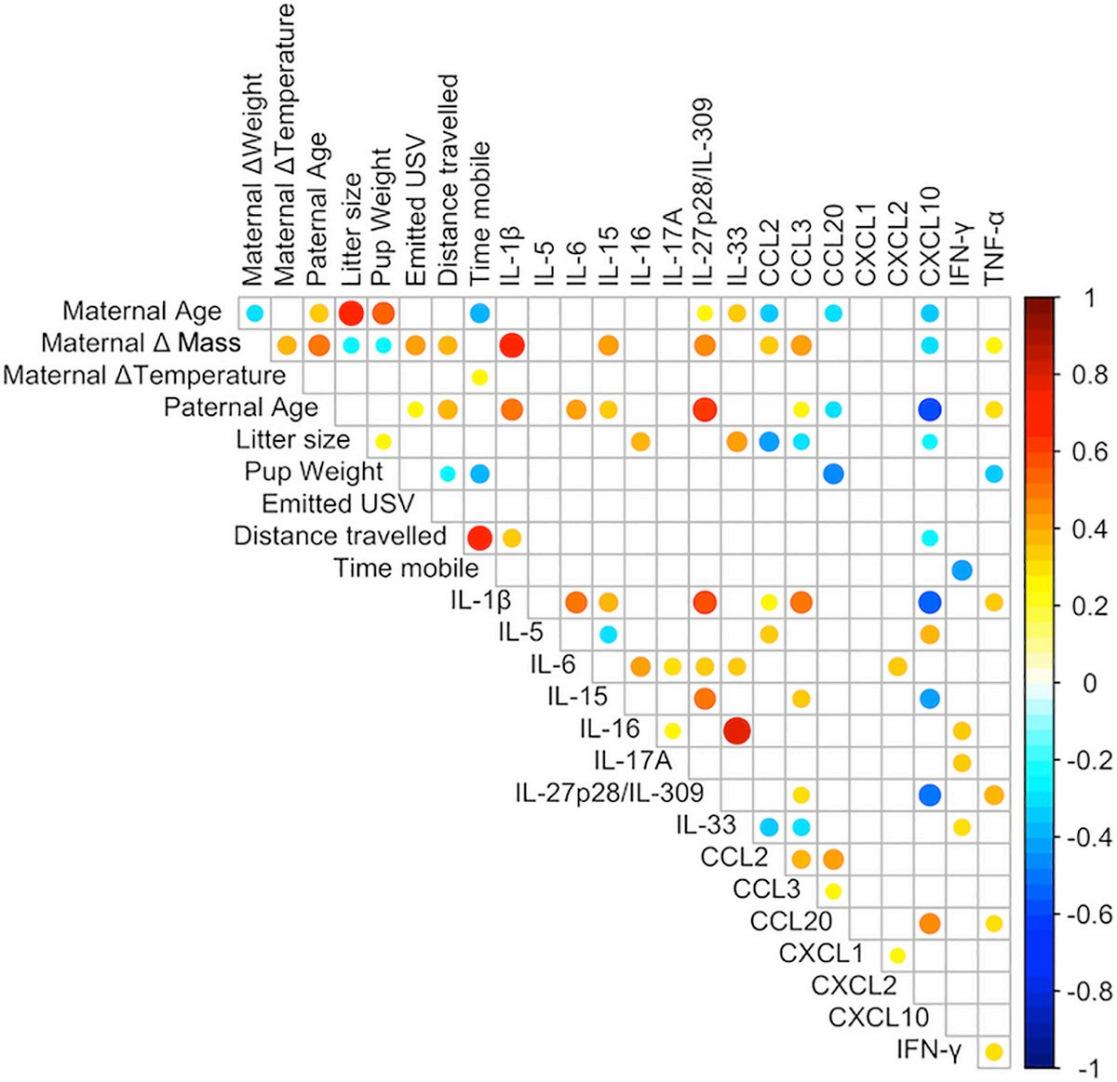
Correlation between numerical variables. Heatmap of pairwise Spearman's rank r correlation coefficients between variable pairs. Spearman’s r coefficients are color-coded and proportional to dot area. Only significant correlations are shown (p < 0.05).

We have collected a total of 26 parental and pup variables in four independent experiments. We only studied male pups, among which 40 and 27 were born from poly(I:C)- and PBS-injected mothers, respectively. Parental variables were maternal and paternal age, multiparity, and the decrease in maternal body temperature and mass 4 days after poly(I:C) injection as surrogates of MIA intensity. Pup’s variables were litter size at P4, body mass at P15, time spent mobile and distance travelled at P13, number of emitted USVs at P8 and serum levels of IFN-γ, IL-1β, IL-5, IL-6, IL-15, IL-16, IL-17A, IL-27p28/IL-30, IL-33, Chemokine C-C motif Ligand (CCL)2, CCL3, CCL20, Chemokine C-X-C motif Ligand (CXCL)1, CXCL2, CXCL10, and TNF-α at P15. Because our sample size was relatively small (n = 67) and the number of variables relatively high (n = 26), the number of events per variable (EPV) was low, i.e. < 10. Further, medium-to-high correlations between the 25 numerical variables of the dataset were observed using the Spearman's r correlation coefficient rank test (Fig 2). These characteristics of our dataset led us to elect a penalized regression framework [31] to model the association between MIA and serum levels of the tested cytokines adjusted for covariates and confounders (Table 1).

**Table 1:** Associations between cytokine levels at P15 and MIA adjusted for confounders and covariates. VIP, mean ORs and 95% CI are shown for the indicated variables. The VIP can be interpreted as the posterior probability of a given variable contribute to the model and is therefore an estimate of the stability of the association. Variables with VIPs above 80% (grey) were considered as stably associated with MIA. Positive (red) and negative (blue) associations are indicated.

	Stage	Variable description	VIP	OR	95% CI
**Parental**	E0.5	Maternal age (weeks)	65.7%	1.139	[1.009,1.411]
**Variables**	E0.5	Multiparity (Yes)	17.2%	1.215	[1.009,2.098]
** **	E13.5	**Maternal** Δ**body mass (g)**	**99.4%**	**0.044**	**[0.001,0.514]**
** **	E12.5	**Maternal** Δ**temperature (°C)**	**92.1%**	**0.217**	**[0.032,0.826]**
** **	E0.5	Paternal age (weeks)	57.3%	1.03	[0.928,1.197]
**Pups'**	P4	**Litter size (nb. pups/litter)**	**93.0%**	**0.334**	**[0.061,0.898]**
**variables**	P15	**Body mass (g)**	**98.7%**	**7.568**	**[1.481,127.402]**
** **	P8	**Number of emitted USV**	**97.0%**	**0.992**	**[0.983,0.998]**
** **	P13	**Distance travelled (m)**	**82.6%**	**0.523**	**[0.183,0.956]**
** **	P13	**Time spent mobile (s)**	**90.8%**	**0.989**	**[0.972,0.999]**
**Cytokines**	P15	CCL2 (pg/ml)	57.4%	0.848	[0.48,1.25]
	P15	CCL3 (pg/ml)	63.9%	0.704	[0.283,1.065]
** **	P15	CCL20 (pg/ml)	43.9%	1.007	[1,1.04]
** **	P15	CXCL1 (pg/ml)	73.2%	0.97	[0.9,1.014]
	P15	CXCL2 (pg/ml)	70.5%	0.867	[0.501,1.096]
** **	P15	**CXCL10** (pg/ml)	**91.5%**	**0.956**	**[0.881,0.995]**
** **	P15	IFN-γ (pg/ml)	59.8%	0.786	[0.069,86.478]
	P15	IL-1-β (pg/ml)	57.2%	1.338	[0.807,2.88]
	P15	**IL-5** (pg/ml)	**85.6%**	**0.823**	**[0.588,0.993]**
	P15	IL-6 (pg/ml)	58.1%	0.965	[0.831,1.134]
** **	P15	**IL-15** (pg/ml)	**85.6%**	**1.035**	**[0.998,1.118]**
** **	P15	IL-16 (pg/ml)	36.3%	1	[1,1]
** **	P15	IL-17A (pg/ml)	53.4%	0.866	[0.007,107.497]
** **	P15	IL-27p28/IL-309 (pg/ml)	74.7%	1.344	[0.975,2.32]
** **	P15	IL-33 (pg/ml)	58.0%	1.052	[1.002,1.175]
** **	P15	**TNF-α** (pg/ml)	**89.7%**	**1.91**	**[1.042,6.41]**

While serum levels of IL-15 and TNF-α at P15 were positively associated with MIA, those of IL-5 and CXCL10 were negatively associated (Table 1). In addition, this analysis allowed for confirming (or not) the results of previous studies (Table 1). More specifically, we found that lower MIA intensity (assessed by a smaller magnitude in the decrease in maternal temperature and body mass at P4), the number of pups per litter at P4, the number of emitted USV at P8, the distance travelled by the pups and the time that they spent mobile at P13 were all negatively associated with MIA (Table 1). In contrast, the pup body mass at P15 was positively associated with MIA (Table 1). Lastly, neither paternal and maternal age, nor multiparity, were associated with MIA (Table 1).

## 4. Discussion

In this study, we have used a penalized regression framework for identifying cytokines at P15 associated with MIA after adjustment for parental and pup covariates and confounders. We found that serum levels of CXCL10 and IL-5 levels were positively associated with MIA while IL-15 and TNF- α levels were negatively associated. None of the other cytokines were associated with MIA. To our knowledge, nothing is known on the impact of MIA on cytokine levels in the progeny during the early postnatal period. Nevertheless, TNF-α levels were found to be increased in serum, placenta and amniotic fluid of pregnant dams immediately after poly(I:C) injection, but not in the foetal brain, nor in the offspring’s serum or brain at P1, P7 or P14 [1, 32, 33]. IL-5 levels were reduced in the cortex and hippocampus of MIA offspring at P7 and in the hippocampus at P14, but not in the serum [1]. While association does not imply causation, our results suggest that changes in TNF-α, IL-15, IL-5 and CXCL10 levels during the postnatal period could contribute to MIA-induced behavioural alterations in mice. In agreement with this latter hypothesis, several *in vitro* and *in vivo* studies have suggested that these cytokines could influence neurodevelopment. First, TNF- α is expressed early in brain development [1] and regulates neurogenesis, synaptogenesis and synaptic scaling in mice [34–37]. Second, IL-15 regulates neural stem cells proliferation and influences behaviour [38, 39]. Further, TNF-α stimulates nuclear export and secretion of IL-15 [40], suggesting an interplay between IL-15 and TNF-α signaling. Lastly, IL-5 is required for neuronal differentiation of hippocampal progenitors [41] and CXCL10 regulates hippocampal synaptic plasticity [42]. On a related topic, a meta-analysis showed that blood levels of TNF-α are higher in ASD patients compared to healthy controls [43]. These data collectively suggest that abnormally elevated levels of TNF-α and IL-15 in conjunction with reduced CXCL10 and IL-5 could perturb normal neurodevelopmental processes and contribute to early behavioural alterations in MIA offspring.

As for confounders and covariates, several of the associations that we have identified had already been observed by others using univariate statistical analysis. For example, most authors have found that MIA induces decreases in maternal body mass and temperature [18, 19] as well as a reduction in the number of pups per litter [18]. In the adult progeny, a robust phenotype induced by MIA was hypolocomotion, as demonstrated by less time being mobile and shorter distance travelled [15, 44]. We show here that MIA pups also present hypolocomotion at early postnatal stages. Less consistent results were obtained for the pup’s body mass during the postnatal period and number of USVs. For example, some authors have found that pups born from poly(I:C)-injected mothers exhibited decreased body mass compared to control animals [45, 46], while others found that these animals had increased fat mass when adults [47]. In agreement with this latter study, we have found that an increased body mass at P15 was associated with MIA after adjustment for confounders and covariates. As for emitted USV, Choi and his co-workers have found increased numbers of USVs produced by MIA pups [16, 48]. In contrast, most authors have observed that both the number and the duration of USV was reduced in mice born from poly(I:C)-injected mothers [49, 50] as well as in several genetic models of ASD [21]. In line with these latter studies we have found that the number of USV at P8 was negatively associated with MIA. USV are produced by pups in response to isolation from the mother, hunger and thermal changes, which call for maternal care: reduced number of USVs can therefore be interpreted as defects in the pup’s attachment behaviour [21]. Our results are therefore expected for a model of ASD.

In contrast to standard univariate analysis, which allowed for comparing variables between groups independently of each other, variables can be analysed altogether using MV statistical methods. Most importantly, MV statistical methods allowed for taking confounders and covariates into account. In statistics, confounders are defined as covariates ancillary to the dependent or independent variables of interest, which can, if not considered, contribute to suggest an effect where there is none or to hide a true effect. MV statistical approaches could prove particularly useful when inter-individual variability within each group is relatively large in models presenting complex inter-dependent phenotypes. In this case, MV methods may allow for performing a statistical ‘‘correction” when all individuals have been assessed for several variables known or believe to interact with each other. The present study underlines the importance of extending the use of MV statistics to further study ASD models, in particular environmentally induced models presenting complex inter-dependent phenotypes.

## Supporting information

S1 Table (XLSX)
